# Prevalence of Mental Health Symptoms and Recommendations in a Cohort of Elite Paralympic versus Olympic Athletes: The Sport Mental Health Assessment Tool 1 (SMHAT-1) in Combination with Clinical Intake Interviews

**DOI:** 10.5114/jhk/204828

**Published:** 2025-09-23

**Authors:** Wojciech Waleriańczyk, Jarosław Krzywański, Katarzyna Wójcik, Grzegorz Lisek, Katarzyna Konopka, Hubert Krysztofiak, Piotr Maleszka, Małgorzata Sławińska, Artur Poczwardowski

**Affiliations:** 1 Department of Social Sciences, Institute of Sport—National Research Institute, Warsaw, Poland.; 2 National Centre for Sports Medicine, Warsaw, Poland.; 3 Department of Rehabilitation, Józef Piłsudski University of Physical Education, Warsaw, Poland.; 4 Pedagogy and Psychology Department, Józef Piłsudski University of Physical Education, Warsaw, Poland.; 5 Faculty of Psychology, SWPS University of Social Sciences and Humanities, Warsaw, Poland.; 6 Mossakowski Medical Research Institute, Polish Academy of Sciences, Warsaw, Poland.; 7 Mazovian Specialist Health Center, Pruszków, Poland.; 8 Graduate School of Professional Psychology, University of Denver, Denver, USA.

**Keywords:** depression, anxiety, eating disorders, sleep disorders, psychological distress

## Abstract

Empirical research regarding the prevalence of mental health symptoms in Paralympic athletes is scarce. We aimed to evaluate the prevalence of mental health concerns in elite Paralympic athletes based on their scores in the IOC Sport Mental Health Assessment Tool 1 (SMHAT-1) and the subsequent in-person clinical intake interviews. In doing so, we also compared the prevalence of mental health symptoms and recommendations in the cohorts of Paralympic and Olympic athletes. We administered the SMHAT-1 (a state-of-the-art tool comprising screeners of psychological distress, depressive and anxiety symptoms, sleep disturbances, alcohol and other substances misuse, and disordered eating) during the routine biannual medical check-ups for all Paralympic athletes at the National Centre for Sports Medicine in Poland. Subsequently, all Paralympic athletes were interviewed by qualified sport psychologists who further evaluated the athletes’ mental health and provided recommendations. A total of 137 Paralympic athletes (87 males, 50 females) participated in the study three to eight months prior to the 2024 Paris Paralympic Games; 61% scored above the triage threshold for psychological distress. Based on the subsequent mental health evaluation, 75.2% of Paralympic athletes required no mental health action, 18.3% were advised psychoeducation or sport psychologist consultation, while 7.3% were referred to a mental health specialist—a psychotherapist and/or a psychiatrist. The proportion of mental health recommendations did not significantly differ between Paralympic and Olympic athletes. The SMHAT-1 proved a valuable basis for the subsequent brief clinical interviews, substantially elevated the efficacy of mental health evaluation, and aided in raising mental health literacy in Paralympic athletes.

## Introduction

Athletes’ mental health has lately become the central feature of many research articles (Anderson et al., 2023; Rice et al., 2020; Waleriańczyk et al., 2025), systematic reviews (Gouttebarge et al., 2019; Pichler et al., 2023), and position stands (Gouttebarge et al., 2021; Moesch et al., 2018; Schinke et al., 2024). In contrast, the mental health of Paralympic athletes (i.e., adaptive athletes competing in sports included in the Paralympic Games) has received comparatively less attention. Even though multiple reviews and position stands underscore the need for comprehensive assessments of Paralympic athletes’ mental health and thorough comparisons with Olympic athletes (Henriksen et al., 2020; Olive et al., 2021, 2022; Schinke et al., 2024; Swartz et al., 2019), empirical research on the subject remains limited. The recent attempts to study mental health in Paralympic athletes include, for example, two cohort studies comparing the prevalence of a broader spectrum of mental health symptoms in Olympic and Paralympic athletes (Anderson et al., 2023; Olive et al., 2021), and three prospective longitudinal studies with brief weekly measurements of depressive and anxiety symptoms (Bentzen et al., 2022; Meidl et al., 2024a, 2024b).

### Prevalence of Mental Health Symptoms in Paralympic Athletes

In a recent study involving 97 Paralympic athletes from Team USA, 9.3% were positively screened for anxiety symptoms, 5.2% for depressive symptoms, 3.1% for suicidal ideation, 10.3% for sleep disturbances, 33% for alcohol misuse, 0% for substance abuse, and 24% for eating disorders (Anderson et al., 2023). These results were corroborated by longitudinal research, where the average prevalence of depressive and anxiety symptoms in Para athletes was close to 6% (Meidl et al., 2024b). What is important from a diagnostic standpoint, almost 40% of Para athletes monitored over 10 months were positively screened for mental health symptoms at least once (Meidl et al., 2024a), while almost 15% of participants were referred to a mental health specialist at least once over 28 months of monitoring (Meidl et al., 2024b). Importantly, the reported mental health concerns were not limited to anxiety and depression symptoms highlighting the need for a broader approach to assessing mental health in Paralympic athletes (Meidl et al., 2024b).

### Comparisons of Mental Health Symptoms between Paralympic and Olympic Athletes

The prevalence of mental health symptoms in the general population is higher among people with disabilities compared to non-disabled individuals (Watson et al., 2014). Additionally, athletes with disabilities (i.e., adaptive athletes) face unique stressors, barriers, and added challenges (Bentzen et al., 2022; Jaarsma et al., 2014; Miller et al., 2024). Against this backdrop, previous research has hypothesized that the prevalence of mental health symptoms should be higher in Paralympic compared to Olympic athletes. Indeed, Anderson et al. (2023) reported slightly higher mean scores for most mental health symptoms in Paralympic compared to Olympic athletes from Team USA. However, these differences were not statistically significant. This pattern, i.e., higher mean scores in Paralympic athletes but no statistically significant differences, was replicated in a study involving 110 Australian para-sport athletes, with a caveat that para-athletes reported lower alcohol use (Olive et al., 2021). In summary, the question whether the prevalence of mental health symptoms differs between Paralympic and Olympic athletes warrants further investigation. Especially studies with higher statistical power (employing measures of high reliability and validity in large samples) could deepen our understanding of the similarities and differences between elite adaptive and able-bodied athletes (Olive et al., 2021; Swartz et al., 2019). Recent research demonstrated that mental health assessment in Olympic athletes can be substantially enhanced with the inclusion of brief clinical intake interviews (Waleriańczyk et al., 2025). However, this approach has yet to be replicated in adaptive athletes.

### Sport Mental Health Assessment Tool 1 in Combination with Brief Clinical Intake Interviews

The necessity for a more comprehensive approach to mental health assessment has been recognized by the International Olympic Committee and was embodied in their recently developed Sport Mental Health Assessment Tool 1 (SMHAT-1) (Gouttebarge et al., 2021). The SMHAT-1 consists of three steps. Athletes who score above the cut-off in the triage for psychological distress (Athlete Psychological Strain Questionnaire; APSQ; Rice et al., 2020) complete six additional screeners of mental health symptoms. Apart from the measures of anxiety (General Anxiety Disorder; GAD-7; Spitzer et al., 2006) and depressive symptoms (Patient Health Questionnaire-9; PHQ-9; Kroenke et al., 2001), step two includes measures of sleep disturbances (Athlete Sleep Screening Questionnaire; ASSQ; Bender et al., 2018; Samuels et al., 2016), alcohol misuse (Alcohol Use Disorders Identification Test Consumption; AUDIT-C; Bradley et al., 2007; Bush et al., 1998), drug misuse (a version of Cutting down, Annoyance by criticism, Guilty feeling and Eye openings; CAGE-AID; Couwenbergh et al., 2009), and eating disorders (Brief Eating Disorders in Athletes Questionnaire; BEDA-Q; Martinsen et al., 2014). Lastly, in step three, athletes who were positively screened for mental health symptoms undergo further clinical assessment, while those who did not are either further monitored or participate in brief interventions (for a detailed description; Gouttebarge et al., 2021).

To further enhance the assessment of mental health in elite sport, two modifications of the SMHAT-1 procedure have been recently proposed. First, screeners from step two are administered to all athletes, regardless of their score in step one, allowing for a more comprehensive description of their mental health (Anderson et al., 2023; Waleriańczyk et al., 2025). Second, the procedure is extended with a brief clinical intake interview conducted by sport psychologists, where scores from the SHMAT-1 serve as a starting point. This provides the psychologist with an opportunity to ask follow-up questions, listen to the athlete discussing their struggles, and assess the severity and persistence of the mental health symptoms reported. Following the interview athletes receive one of three mental health recommendations based on the International Society of Sport Psychology position stand (Schinke et al., 2024), accompanied by the most appropriate course of mental health action: 1) no/low symptoms—no action required; 2) occasional/short-term symptoms or concerning issues—psychoeducation and/or a consultation with a sport psychologist advised; 3) high and persistent mental health concerns—referral to a psychotherapist and/or psychiatrist. The inclusion of these two modifications has been shown to elevate the diagnostic effectiveness of the SMHAT-1 in a cohort of Olympic athletes (Waleriańczyk et al., 2025). However, neither its effectiveness nor the sensitivity and specificity of step one (APSQ) has been evaluated in relation to mental health recommendations in Paralympic athletes.

### The Present Study

In the present study, our main aims were to 1) assess the prevalence of mental health concerns in the cohort of Polish elite Paralympic athletes based on the SMHAT-1 and the subsequent brief clinical intake interviews, conducted by qualified sport psychologists, and 2) compare the prevalence of mental health concerns and the subsequent mental health recommendations between the cohorts of Polish Paralympic and Olympic athletes participating in national-team-level mental health monitoring programme (Waleriańczyk et al., 2025). In doing so, we also provided the first in-depth analysis of sensitivity, specificity, and predictive value of the SMHAT-1 triage (i.e., APSQ) in predicting subsequent mental health evaluations in Paralympic athletes.

## Methods

### Study Design, Procedure, and Data Collection

The study was conducted in person at the National Centre for Sports Medicine in Poland, during athletes’ biannual medical check-ups from November 2023 to June 2024. Participation was confidential and not mandatory. The procedure and the study design were identical as in the Polish Olympic athletes study (Waleriańczyk et al., 2025) to allow comparing the results from both groups. After accepting the study terms, Para athletes completed SMHAT-1 forms one and two and, subsequently, participated in a brief (10–15 min) clinical intake interview based on their scores in the SMHAT-1.

The clinical intake interviews were conducted by a team of four qualified sport psychologists with previous clinical experience; sport psychologists followed an interview structure and guidelines formulated and agreed upon by the research team led by the first author. All research team members working with Paralympic athletes, i.e., sport psychologists, psychotherapists, and a psychiatrist, participated in supervision (Poczwardowski et al., 2023). The mental health recommendations are described in Table 1, while a more detailed description of the procedure and the content of the interviews is available at the Open Science Framework (OSF) under the following link: https://osf.io/p5he2

**Table 1:** The psychologists’ recommendations for different evaluations of mental health status and brief descriptions of examples of essential information gathered during the clinical intake interviews

Evaluation of the athlete’s mental health status	Action recommended by the psychologist	Example case
Sound mental health, where one is asymptomatic, with broad-based optimized functioning	No symptoms—no particular mental health-related action required	Psychological distress slightly above the cut-off, but no other positive screenings. The interview shows no maladaptive coping mechanisms, athlete’s general well-being seems to be high.
Concerning issues that do not meet diagnostic criteria yet warrant support	Psychoeducation and/or referral to a sport psychologist required	Positive screening for alcohol and drugs misuse. The interview shows the need for adjusting to the new team of more experienced athletes with alcohol and drugs being the way of achieving closer connections with the team.
Distress levels, occasional or short-term symptoms (understood as normal in relation to demands)	Psychoeducation and/or referral to a sport psychologist required	High psychological distress, moderate anxiety symptoms, and sleep disturbances. The interview adds important context—the anxiety and sleep disturbances are related to upcoming important exams and no history of mental health symptoms is present.
Active mental illness/high and persistent mental health concerns	Referral to a mental health specialist (a psychotherapist and/or psychiatrist)	Positively screened for psychological distress, depressive symptoms, and disordered eating. The interview shows signs of persistent anhedonia, recurring depressive symptoms, and maladaptive coping mechanisms.

Note. Mental health status categories based on the International Society of Sport Psychology position stand (Schinke et al., 2024). Table adapted from Waleriańczyk et al. (2025)

### Participants and Sample Size Justification

A total of 137 elite Polish Paralympic athletes (87 males, 50 females), aged 19 to 62 (M = 35.99, SD = 10.27) completed the SMHAT-1 and the subsequent clinical intake interview. Since this represented over 90% of the Polish elite Paralympic cohort, the results of this study provide population-level insights, and by eliminating the sampling variability serve as a sufficient sample size justification (Lakens, 2022). As a way of benchmarking, ultimately, the Polish Paralympic team for the Paris 2024 Paralympic Games consisted of 84 Para athletes.

### Statistical Analysis

***Preliminary Analyses*.** First, we calculated Cronbach’s alphas to test the reliability of all measures. Furthermore, we tested whether the assumptions or normality of the distribution and homogeneity of the variance were not violated, to decide whether parametric or non-parametric tests should be used. As the distributions were non-normal in all measures (except for the APSQ, where the result was on the margins of the *p* value < 0.05), we used the Mann-Whitney U Test (Nachar, 2008) to compare mean scores between males and females.

***Prevalence of Mental Health Symptoms and Recommendations*.** Second, we computed descriptive statistics for all questionnaires and the frequencies of psychologists’ recommendations for all participants and separately for males and females.

***Comparisons between Paralympic and Olympic Athletes*.** Third, we used the Mann-Whitney U Test to compare mean scores in mental health symptoms between Paralympic and Olympic athletes, and the chi-square test of independence to compare the proportions of mental health recommendations in Paralympic and Olympic athletes. In doing so, we used the database from the study of the Polish Olympic athletes cohort (Waleriańczyk et al., 2025), available as a supplementary material in the referenced study (data available here: https://bjsm.bmj.com/highwire/filestream/136645/field_highwire_adjunct_files/1/bjsports-2024-108919supp002_data_supplement.csv).

***APSQ Performance in Predicting Mental Health Symptoms and Recommendations*.** Fourth, we constructed confusion matrices for all participants and separately for males and females to calculate the specificity and sensitivity of the APSQ in relation to 1) each of the SMHAT-1 measures, 2) any SMHAT-1 positive screening, and 3) the psychologists’ recommendations. We also calculated the areas under the curve (AUC) for the receiver operator characteristic (ROC) curves for the triage against different benchmarks to determine the optimal sensitivity and specificity based on the Youden index (Böhning et al., 2008). All analyses were performed using SPSS. All figures were created in Python.

## Results

### Preliminary Analyses

***Bivariate Correlations***. Psychological distress was significantly and positively correlated with anxiety symptoms, depressive symptoms, and sleep disturbances, but not with eating disorders and alcohol and substance misuse (Table 2). Anxiety symptoms and depressive symptoms showed high intercorrelation and were also associated with sleep disturbances, but not with eating disorders and alcohol and substance misuse. Athletes’ age was positively correlated with alcohol misuse and negatively correlated with depressive symptoms.

**Table 2:** Descriptive statistics by sex, reliability, and bivariate correlations between mean scores in all questionnaires

Questionnaire (Reliability α)	All athletes mean ± SD range	Female mean ± SD range	Male mean ± SD range	1.	2.	3.	4.	5.	6.	7.
1. APSQ (0.77)	17.82 ± 4.83 (10–33)	17.64 ± 4.72 (11–33)	17.92 ± 4.91 (11–33)	-						
2. GAD-7 (0.88)	3.80 ± 3.95 (0–21)	4.18 ± 3.72 (0–19)	3.58 ± 4.09 (0–21)	0.50**	-					
3. PHQ-9 (0.79)	4.02 ± 3.64 (0–22)	4.40 ± 2.19* (0–14)	3.79 ± 4.00 (0–22)	0.45**	0.70**	-				
4. ASSQ (0.68)	6.15 ± 2.87 (1–16)	6.26 ± 3.10 (2–16)	6.08 ± 2.75 (1–14)	0.32**	0.32**	0.42**	-			
5. AUDIT-C (0.65)	1.62 ± 1.56 (0–7)	1.30 ± 0.97 (0–4)	1.80 ± 1.79 (0–7)	0.17	0.01	0.11	0.12	-		
6. CAGE-AID (0.65)	0.12 ± 0.47 (0–4)	0.06 ± 0.24 (0–1)	0.15 ± 0.56 (0–4)	0.08	0.03	0.06	0.15	0.16	-	
7. BEDA-Q (0.64)	2.32 ± 2.70 (0–13)	2.44 ± 2.93 (0–13)	0.15 ± 0.56 (0–12)	0.11	0.12	0.16	0.09	–0.03	0.05	-
8. Age	35.99 ± 10.27 (19–62)	33.18 ± 8.80 (19–48)	37.60 ± 10.74 (19–62)	–0.05	–0.09	–0.22*	0.09	0.02	0.20*	0.04

Note. Z (p) - the Z value for the Mann-Whitney U-test for the comparison between the sexes and the accompanying p-value. APSQ: Athlete Psychological Strain Questionnaire; GAD-7: General Anxiety Disorder-7; PHQ-9: Patient Health Questionnaire-9; ASSQ: Athlete Sleep Screening Questionnaire; AUDIT-C: Alcohol Use Disorders Identification Test Consumption; CAGE-AID: Cutting Down, Annoyance by criticism, Guilty feeling and Eye openings adapted to include drugs; BEDA-Q: Brief Eating Disorders in Athletes Questionnaire; ** significant at p < 0.001; * significant at p < 0.05

***Comparison by Sex***. Depressive symptoms were significantly higher in female athletes (z = −2.13; *p* = 0.033). No other significant sex differences were found in the mean scores for psychological distress (–0.679; *p* = 0.497), anxiety symptoms (z = –1.457; *p* = 0.145), sleep disturbances (z = –0.092; *p* = 0.926), alcohol misuse (z = –0.702; *p* = 0.482), drugs misuse (z = –0.110; *p* = 0.913), and disordered eating (z = –0.831; *p* = 0.406). Even though the differences were not significant, it should be noted that female Paralympic athletes scored slightly higher in anxiety symptoms, and disordered eating (Table 2). There were no significant sex differences in the frequency of receiving the ‘no action required’ (z = −1.47, *p* = 0.142), ‘psychoeducation and/or a consultation with a sport psychologist advised’ (z = −1.32, *p* = 0.188), and ‘referral to a psychotherapist and/or psychiatrist’ (z = −0.24, *p* = 0.812) recommendation.

### Prevalence of Mental Health Symptoms and Recommendations

**Figure 1:**
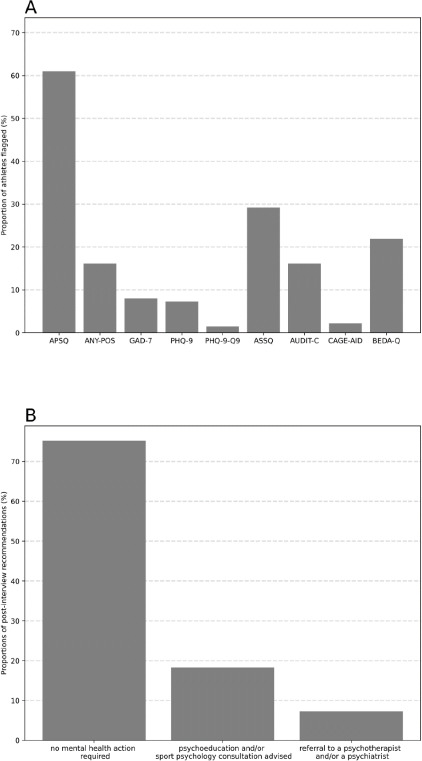
Proportions of athletes flagged in each questionnaire, using the official thresholds from the SMHAT 1 (panel A), and proportions of athletes with different recommendations following the brief clinical intake interview based on the results from the SMHAT 1 (panel B). *APSQ: Athlete Psychological Strain Questionnaire; GAD-7: General Anxiety Disorder-7; PHQ-9: Patient Health Questionnaire-9; ASSQ: Athlete Sleep Screening Questionnaire; AUDIT-C: Alcohol Use Disorders Identification Test Consumption; CAGE-AID: Cutting Down, Annoyance by criticism, Guilty feeling and Eye openings adapted to include drugs; BEDA-Q: Brief Eating Disorders in Athletes Questionnaire*

***Mental Health Symptoms*.** A total of 83 out of 137 participating athletes scored above the triage threshold (i.e., 17 points or higher in the APSQ); 22 athletes scored above the advised cut-off points in at least one of the screeners (triage excluded), 10 athletes in one screener, 8 in two, and 4 in three screeners. More specifically, 11 athletes were flagged for anxiety symptoms, 8 for depressive symptoms, 2 for suicidal ideation (in both cases of suicidal ideation, the SAD PERSONS scale (Patterson et al., 1983) was employed by the psychologists to assess the need for immediate action, however, in both cases, it was not required). Athletes were referred to a psychiatrist and were in treatment. Most recent updates confirm improvements in mental health), 3 for drug misuse, 22 for alcohol misuse, 40 for sleep disturbances, and 30 for eating disturbances (Figure 1).

**Figure 2:**
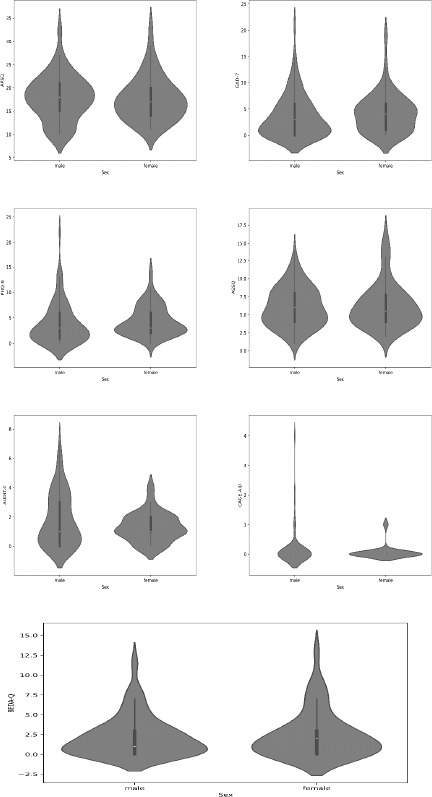
Violin plots showing the distributions of the scores in all questionnaires by gender. APSQ: Athlete Psychological Strain Questionnaire; GAD-7: General Anxiety Disorder-7; PHQ-9: Patient Health Questionnaire-9; ASSQ: Athlete Sleep Screening Questionnaire; AUDIT-C: Alcohol Use Disorders Identification Test Consumption; CAGE-AID: Cutting Down, Annoyance by criticism, Guilty feeling and Eye openings adapted to include drugs; BEDA-Q: Brief Eating Disorders in Athletes Questionnaire

***Mental Health Recommendations*.** Furthermore, 103 athletes received the ‘no action required’ recommendation, 25 the ‘psychoeducation and/or a consultation with a sport psychologist advised’ recommendation, and 10 the ‘referral to a psychotherapist and/or psychiatrist’ recommendation. One athlete received two simultaneous recommendations.

### Comparisons of Mental Health Symptoms and Recommendations between Paralympic and Olympic Athletes

***Mental Health Symptoms*.** Para athletes scored significantly lower in psychological distress (z = −3.93, *p* < 0.001) and significantly higher in sleep disturbances (z = 2.54, *p* = 0.011) compared to Olympic athletes. No significant differences were found for anxiety symptoms (z = −0.99, *p* = 0.324), depressive symptoms (z = −1.52, *p* = 0.128), alcohol misuse (z = −1.89, *p* = 0.058), drugs misuse (z = −0.68, *p* = 0.496), and disordered eating (z = −0.32, *p* = 0.752).

***Mental Health Recommendations*.** No significant differences were found between Paralympic and Olympic athletes in the frequency of receiving the ‘no action required’ (χ^2^(1) = 2.75, *p* = 0.097), ‘psychoeducation and/or a consultation with a sport psychologist advised’ (χ^2^(1) = 1.82, *p* = 0.177), and ‘referral to a psychotherapist and/or psychiatrist’ (χ^2^(1) = 0.73, *p* = 0.393) recommendations.

### Diagnostic Accuracy and Threshold Evaluation of the Triage (APSQ) in Relation to Mental Health Symptoms and Recommendations

***False Negative Rates (Miss Rates)*.** The global FNR for the SMHAT-1 (i.e., athletes who scored below the triage cut-off, but were positively screened on at least one other measure) was 27.3%. The FNR for specific questionnaires ranged from 0% (GAD-7, PHQ-9, PHQ-9.Q9) to 66.7% (CAGE-AID). Regarding mental health recommendations, the FNR for athletes who scored below the triage but required any mental health action (i.e., received the ‘psychoeducation and/or a consultation with a sport psychologist advised’ or the ‘referral to a psychotherapist and/or psychiatrist’ recommendation) was 40%, while the FNR for those referred to a mental health specialist was 0% (Table 3).

**Table 3:** Confusion matrices, FNRs, FPRs, TNRs, and TPRs and the area under the curve (AUC) for the receiver operator characteristic curve (ROC) for all athletes

	Threshold	APSQ score		AUC	Rates (%)			
		<17	≥17		FNR	FPR	PPV	NPV
GAD-7	<10	54	72	0.80	0	57.1	13.3	100
	≥10	0	11					
PHQ-9	<10	54	75	0.82	0	58.1	9.6	100
	≥10	0	8					
PHQ-9 Q9	<1	54	81	0.77	0	60	2.4	100
	≥1	0	2					
ASSQ	<8	42	55	0.65	30	56.7	33.7	77.8
	≥8	12	28					
AUDIT-C	<4 (M), <3(F)	48	67	0.60	27.3	58.3	19.3	88.9
	≥4 (M), ≥3 (F)	6	16					
CAGE-AID	<2	52	82	0.49	66.7	61.2	1.2	96.3
	≥2	2	1					
BEDA-Q	<4	46	61	0.64	26.7	57.0	26.5	85.2
	≥4	8	22					
Any positive SMHAT 1 screening	No	48	67	0.60	27.3	58.3	19.3	88.9
	Yes	6	16					
Mental health action advised	No	44	68	0.53	40	60.7	18.1	81.5
	Yes	10	15					
Referral to mental health specialist	No	54	73	0.87	0	57.5	12	100
	Yes	0	10					

Note. Miss rate: FNR = FN/(FN+TP); False alarm rate: FPR = FP/(FP+TN); Specificity: TNR = TN/(TN+FP); Sensitivity: TPR = (TP/TP+FN); APSQ: Athlete Psychological Strain Questionnaire; GAD-7: General Anxiety Disorder-7; PHQ-9: Patient Health Questionnaire-9; ASSQ: Athlete Sleep Screening Questionnaire; AUDIT-C: Alcohol Use Disorders Identification Test Consumption; CAGE-AID: Cutting Down, Annoyance by criticism, Guilty feeling and Eye openings adapted to include drugs; BEDA-Q: Brief Eating Disorders in Athletes Questionnaire

***False Positive Rates (False Alarms)*.** The global FPR for the SMHAT-1 (i.e., athletes who scored above the triage cut-off but were not positively screened on any other measure) was 58.3%, ranging from 57% (BEDA-Q) to 61.2% (CAGE-AID). Females had lower FPRs for all screenings. Regarding mental health recommendations, the FPR for athletes who scored above the triage cut-off but required no mental health action was 60.7%, and for those not referred to a mental health specialist it was 57.5%. Both FPRs were slightly lower in females (Tables 4 and 5).

**Table 4:** Confusion matrices, FNRs, FPRs, TNRs, and TPRs and the area under the curve (AUC) for the receiver operator characteristic curve (ROC) for female athletes

	Threshold	APSQ score		AUC	Rates (%)			
		<17	≥17		FNR	FPR	PPV	NPV
GAD-7	<10	21	27	0.94	0	56.3	6.9	100
	≥10	0	2					
PHQ-9	<10	21	27	0.99	0	56.3	6.9	100
	≥10	0	2					
PHQ-9 Q9	<1	21	28	0.90	0	57.1	3.4	100
	≥1	0	1					
ASSQ	<8	17	20	0.66	30.8	54.1	31	81
	≥8	4	9					
AUDIT-C	<4 (M), <3(F)	20	26	0.63	25.0	56.5	10.3	95.2
	≥4 (M), ≥3 (F)	1	3					
CAGE-AID	<2	21	29	N/A	N/A	58.0	0	100
	≥2	0	0					
BEDA-Q	<4	18	20	0.66	25.0	52.6	31	85.7
	≥4	3	9					
Any positive SMHAT 1 screening	No	20	26	0.57	25.0	56.5	10.3	95.2
	Yes	1	3					
Mental health action advised	No	16	22	0.53	41.7	57.9	24.1	76.2
	Yes	5	7					
Referral to a mental health specialist	No	21	25	0.96	0	54.3	13.8	100
	Yes	0	4					

Note. Miss rate: FNR = FN/(FN+TP); False alarm rate: FPR = FP/(FP+TN); Specificity: TNR = TN/(TN+FP); Sensitivity: TPR = (TP/TP+FN); APSQ: Athlete Psychological Strain Questionnaire; GAD-7: General Anxiety Disorder-7; PHQ-9: Patient Health Questionnaire-9; ASSQ: Athlete Sleep Screening Questionnaire; AUDIT-C: Alcohol Use Disorders Identification Test Consumption; CAGE-AID: Cutting Down, Annoyance by criticism, Guilty feeling and Eye openings adapted to include drugs; BEDA-Q: Brief Eating Disorders in Athletes Questionnaire

**Table 5:** Confusion matrices, FNRs, FPRs, TNRs, and TPRs and the area under the curve (AUC) for the receiver operator characteristic curve (ROC) for male athletes

	Threshold	APSQ score		AUC	Rates (%)			
		<17	≥17		FNR	FPR	PPV	NPV
GAD-7	<10	33	45	0.76	0	57.7	16.7	100
	≥10	0	9					
PHQ-9	<10	33	48	0.74	0	59.3	11.1	100
	≥10	0	6					
PHQ-9 Q9	<1	33	53	0.59	0	61.6	1.9	100
	≥1	0	1					
ASSQ	<8	25	35	0.63	29.6	58.3	35.2	75.8
	≥8	8	19					
AUDIT-C	<4 (M), <3(F)	28	41	0.57	27.8	59.4	24.1	84.8
	≥4 (M), ≥3 (F)	5	13					
CAGE-AID	<2	31	53	0.47	66.7	63.1	1.9	93.9
	≥2	2	1					
BEDA-Q	<4	28	41	0.63	27.8	59.4	24.1	84.8
	≥4	5	13					
Any positive SMHAT 1 screening	No	28	41	0.63	27.8	59.4	24.1	84.8
	Yes	5	13					
Mental health action advised	No	28	46	0.55	38.5	62.2	14.8	84.8
	Yes	5	8					
Referral to a mental health specialist	No	33	48	0.80	0	59.3	11.1	100
	Yes	0	6					

Note. Miss rate: FNR = FN/(FN+TP); False alarm rate: FPR = FP/(FP+TN); Specificity: TNR = TN/(TN+FP); Sensitivity: TPR = (TP/TP+FN); APSQ: Athlete Psychological Strain Questionnaire; GAD-7: General Anxiety Disorder-7; PHQ-9: Patient Health Questionnaire-9; ASSQ: Athlete Sleep Screening Questionnaire; AUDIT-C: Alcohol Use Disorders Identification Test Consumption; CAGE-AID: Cutting Down, Annoyance by criticism, Guilty feeling and Eye openings adapted to include drugs; BEDA-Q: Brief Eating Disorders in Athletes Questionnaire

***Positive Predictive Value (PPV)*.** The global PPV (i.e., the probability that an athlete who scored above the triage cut-off would be positively screened in any other screener) was 19.3% and ranged from 1.2% for CAGE-AID to 33.7% for the ASSQ. Regarding mental health recommendations, the probability of requiring mental health action after being positively screened in triage was 18.1%, while the probability of requiring referral was 12%.

***Negative Predictive Value (NPV)*.** The global NPV (i.e., the probability that an athlete below the triage cut-off would not be positively screened in any other screener) was 88.9% and ranged from 77.8% for ASSQ to 100% for GAD-7, PHQ9, and PHQ9.Q9. Regarding mental health recommendations, the probability of not requiring mental health action after scoring below the cut-off in triage was 88.9%, while the probability of not requiring referral was 100%.

***Recalculating the APSQ Threshold***. The AUC for the ROC curves (with the ≥17 triage threshold) for the SMHAT-1 measures provided diversified values depending on the analyzed outcome and ranged from 0.49 (CAGE-AID)to 0.82 (PHQ-9). The AUC for the ’mental health specialist referral’ was the highest value across all the outcomes (0.87), however, it was rather low for the ‘mental health action advised’ (0.53) (Table 3).

Only the lowest possible value in the triage (i.e., 10) allowed for predicting all subsequent positive screenings in at least one of the SMHAT-1 measures. However, with referral to a mental health specialist as the main benchmark, a score of ≥19 allowed predicting all cases in which athletes needed a referral and was accompanied by the highest Younden’s index of 0.63 and 41.6% of athletes being positively screened in triage (Table 6).

**Table 6:** FNRs, FPRs, PPVs, and NPVs for different cut-off values of the SMHAT-1 triage against mental health referral as the benchmark

Threshold	FNR	FPR	PPV	NPV	Youden’s index
≥17	0	57.5	12	100	0.43
≥18	0	47.2	14.3	100	0.53
≥19	0	37	17.5	100	0.63
≥20	30	29.9	15.6	96.7	0.40
≥21	30	22	20	97.1	0.48
≥22	30	16.5	25	97.2	0.53
≥23	40	10.2	31.6	96.6	0.50
≥24	57.1	3.3	60	93.7	0.40
≥25	58.3	4	50	94.5	0.38

Note. Miss rate: FNR = FN/(FN+TP); False alarm rate: FPR = FP/(FP+TN); Specificity: TNR = 100 – FPR; Sensitivity: TPR = 100 – FNR; Positive predictive value: PPV = TP/(TP+FP); Negative predictive value: NPV = TN/(TN+FN); Youden’s index = TPR + TNR – 1; higher values reflect better diagnostic effectiveness

## Discussion

In the current study, we provided the first report on the prevalence of mental health symptoms in a cohort of Polish Paralympic athletes, based on the SMHAT-1 scores and the subsequent brief clinical intake interviews. Furthermore, we demonstrated how the mental health concerns and the subsequent mental health recommendations differed between the Polish Paralympic and Olympic athletes participating in the newly introduced national team-level mental health monitoring programme. We also discussed how the new data on the performance of the triage could be used to optimize the mental health screening process in Para athletes.

### Mental Health of Polish Elite Paralympic Athletes

***Prevalence of Mental Health Symptoms*.** Out of the 137 Paralympic athletes, 16.1% were positively screened in at least one of the screeners, 7.3% in one, 5.8% in two, and 2.9% in three screeners. In particular, 8% were positively screened for anxiety symptoms, 7.3% for depressive symptoms, 1.5% for suicidal ideation, 2.2% for drug misuse, 16.1% for alcohol misuse, 29.2% for sleep disturbances, and 21.9% for eating disorders. Female athletes were more frequently screened for depressive symptoms (no other significant sex differences were noted in the current sample). These rates are very similar to those reported in previous research (Bentzen et al., 2022; Meidl et al., 2024a, 2024b; Olive et al., 2021), with the caveat that Polish Paralympic athletes are three times more likely to be positively screened for sleep disturbances and two times less likely to be screened for alcohol misuse than Team USA Paralympic athletes (Anderson et al., 2023).

***Mental Health Recommendations Based on the Clinical Intake Interviews*.** Following the clinical intake interviews based on the SMHAT-1 forms one and two scores, 75.2% of Paralympic athletes were assessed as having sound mental health and required no mental health action. In 18.3% of Paralympic athletes distress levels/symptoms were assessed as occasional, short-term, and normal in relation to demands—they were advised psychoeducation or sport psychologist consultation, while 7.3% were referred to a mental health specialist—a psychotherapist, and/or a psychiatrist. It should be noted that one in four Paralympic athletes required some form of mental health support—from psychoeducation to a referral to a sport psychologist, a psychotherapist or a psychiatrist. This attests to the necessity of including mental health assessment during routine medical check-ups, especially given that the proportion of those in need of mental health support increases over longer time intervals (Meidl et al., 2024b), and may fluctuate depending on the moment of the season (Bentzen et al., 2022).

### Mental Health of Paralympic Compared to Olympic Athletes

***Prevalence of Mental Health Symptoms*.** When assessed using SMHAT-1 measures, Polish Paralympic and Olympic athletes exhibited similar rates of positive screenings, with all except two being slightly less frequent in Paralympic athletes. The two exceptions were anxiety symptoms and sleep disorders, with only the latter difference being substantial and statistically significant. The fact that 29.2% of Paralympic athletes were positively screened for sleep disturbances (compared to 19.9% of Olympic athletes) reflects specific additional challenges of Paralympic athletes (Miller et al., 2024). The clinical intake interviews provided insights into the causes of these differences; in many cases the sleep disturbances were related to more frequent interruptions in sleep due to numbness in different areas of the body, and the need to change the position. Furthermore, athletes with acquired disabilities also reported that in some cases their sleep disturbances were related to traumatic events from the past. This underscores the added value of extending the SMHAT-1 procedure with brief clinical intake interviews.

***Mental Health Recommendations*.** Even though the percentage of athletes that required any mental health action was lower in the present group of Paralympic athletes compared to Olympic athletes (with 9.5% of Olympic athletes referred to a mental health specialist, and 24.2% advised other mental health action) (Waleriańczyk et al., 2025), there were no statistically significant differences between the proportions of Olympic and Paralympic athletes for any of the three mental health recommendations.

***Overall Discussion of the Differences*.** The current study does not support the notion that Paralympic and Olympic athletes significantly differ in the prevalence of mental health symptoms. This contrasts with evidence from the general population where mental health symptoms are higher among people with disabilities (Watson et al., 2014). However, it is in line with two previous cohort studies from the USA and Australia that did not report significant differences between these two groups (Anderson et al., 2023; Olive et al., 2021). Similarly, but regarding psychological skills for performance, sport psychology researchers (Henschen et al., 1992; Martin, 2002) and practitioners (Dieffenbach and Statler, 2012) have been long reporting more similarities than differences between adaptive and able-bodied athletes. Furthermore, there is evidence that suggests a positive impact of sports participation on adaptive athletes’ well-being and resilience, and that the positive effects increase with higher skill levels (Mira et al., 2023). This alludes to the possibility that sport participation may also positively affect adaptive athletes’ mental health.

Previous research has also underscored that adaptive athletes experience a range of additional barriers, stressors, and challenges (both sport-specific and disability-specific) (Bentzen et al., 2022; Miller et al., 2024; Swartz et al., 2019). However, in the current study one of the rare instances where Olympic and Paralympic athletes differed in a statistically significant manner was the level of psychological distress, which was lower in Paralympic athletes. It could be that the additional challenges offer unwanted yet persistent opportunities to train psychological resilience that could not only reduce the perceived difficulty of the daily challenges but also explain the overall lack of differences in ill-being observed in the current study. Furthermore, given that the mean age of Paralympic athletes is substantially higher than that of Olympic athletes and that we found a negative correlation between age and depressive symptoms, perhaps more life experience translates into better coping skills that in turn can support mental health (Taylor and Stanton, 2007) and somewhat counteract the unique stressors and added challenges.

### The Triage Performance

***Positive Screenings for Psychological Distress*.** In the present study, 61% of Paralympic athletes scored above the cut-off point for psychological distress (i.e., APSQ) compared to 37.1% of Paralympic athletes in the Team USA study (Anderson et al., 2023). The fact that Polish Para athletes are almost twice as likely to be screened for psychological distress compared to the Team USA Para athletes may in part stem from the measurement in the Polish sample being conducted in the proximity to the Paralympic Games (three to eight months compared with the 18 months of data collection with the Team USA for the Tokyo and Beijing Games). However, it could also reflect cultural differences, differences in the levels of psychological safety, lower perceived support, and lower financial stability among Polish athletes, especially given that similar differences were found between Polish and American Olympic athletes (Waleriańczyk et al., 2025).

***Optimal Cut-Off Values*.** Overall, the triage cut-off value of ≥17 allowed for a very accurate prediction of athletes who were not positively screened in subsequent measures, did not need any mental health action, and did not require referral (NPVs of 88.9%, 81.5%, and 100%, respectively). However, this was accompanied by rather poor accuracy in predicting those that would subsequently be positively screened in any measure, need any mental health action, and referral (PPVs of 19.3%, 18.1%, and 12%, respectively). This further expands the discussion on the most appropriate cut-off value for the triage (Gouttebarge et al., 2021; Rice et al., 2020; Shannon et al., 2024; Waleriańczyk et al., 2025). In the current sample, with the mental health referral as the benchmark, the cut-off value of ≥19 would be associated with the highest Youden index value but at a rather low PPV. A cut-off value of ≥22 would be associated with the second-highest Youden index, and a higher PPV, while a cut-off value of ≥23 would lead to an increase in PPV but at a slightly higher risk of not identifying those in need of mental health referral. Thus, similarly as in the sample of Polish Olympic athletes (Waleriańczyk et al., 2025), elevating the threshold by at least two points would result in a more efficient procedure with lower FPRs, accompanied by adequate sensitivity (Table 6). However, since the APSQ predicts some mental health symptoms more accurately than others, we recommend using it alongside all subsequent SMHAT-1 measures for a more comprehensive assessment of mental health in adaptive athletes.

### The Added Value of Brief Clinical Intake Interviews Based on the SMHAT-1

The current study highlights that brief clinical intake interviews, based on SMHAT-1 results, provided a valuable foundation for a systematic, national-level mental health monitoring program. Extending the variable-centered approach of standardized questionnaires with the person-centered approach of brief interviews leveraged the advantages of both methods. While standardized questionnaires offer high reliability, ease of administration, and assessment based on cut-off values, brief clinical interviews complemented these strengths by providing a flexible, individualized approach to mental health. The interviews allowed athletes to express their most relevant concerns, facilitated rapport-building for future check-ups, and enabled clarification and deeper contextual understanding of mental health needs. Thus, whenever feasible, we recommend supplementing the SMHAT-1 with brief clinical intake interviews conducted by qualified sport psychologists to enhance the diagnostic effectiveness of mental health monitoring and mental health literacy in adaptive athletes.

### Limitations and Future Directions

Even though we reached almost all elite Paralympic athletes in the Polish cohort and the number of participants in the current study is higher compared to the previous longitudinal (Bentzen et al., 2022; Meidl et al., 2024a, 2024b), and cohort studies (Anderson et al., 2023; Olive et al., 2021), it should be noted that the cohort of Paralympic athletes is substantially smaller compared to the Olympic athletes cohort. Thus, the analyses have less statistical power than in the able-bodied group. Furthermore, the measurement timing (proximity to the Paris 2024 Paralympic Games) may have led to reporting more mental health symptoms (Bentzen et al., 2022; Henriksen et al., 2020; Mountjoy et al., 2023). Alternatively, the method of data collection (in-person, and confidential rather than anonymous) may have led to underreporting of mental health symptoms (Harenberg et al., 2024), which further cautions possible interpretations.

Importantly, even though the SMHAT-1 is a valuable addition to the field, it is only the first attempt in standardized mental health assessment of athletes and it should be further developed. This should include 1) further refinement of the triage to elevate its predictive validity in terms of subsequent positive screenings and mental health recommendations, and 2) further refinement or inclusion of more reliable and valid screeners for eating disorders, alcohol misuse, and drugs misuse. From a more practical perspective, it would also be beneficial to include a measure of well-being; this would allow shifting focus from ill-being to a dual continuum operationalization of mental health. Furthermore, since certain disorders (e.g., body dysmorphia) could be more prevalent among athletes with physical disabilities (Tahir et al., 2024), future research should explore how to best tailor the standardized mental health assessment for adaptive athletes.

## Conclusions

Based on the SMHAT-1 and the subsequent brief clinical intake interviews, one in four elite Polish Paralympic athletes was in need of mental health action and received psychoeducation or was referred to a specialist: a sport psychologist, a psychotherapist or a psychiatrist. As such there were no significant differences in the prevalence of mental health symptoms between the cohorts of Polish Paralympic and Olympic athletes, with a caveat that Paralympic athletes reported more sleep disturbances. The SMHAT-1 has proved to be a useful screening basis for the subsequent clinical intake interviews with elite Paralympic athletes during their routine biannual medical check-ups. Whenever feasible, we recommend supplementing SMHAT-1 forms one and two with a clinical intake interview to enhance diagnostic efficacy. In settings where this is not possible, we recommend using both the APSQ and all subsequent SMHAT-1 measures in all adaptive athletes, to provide a more comprehensive description of their mental health.

## Data Availability

Anonymized database from the project is available here: https://osf.io/pt9hb
